# Single nucleotide polymorphisms of *NR3C1* gene and recurrent depressive disorder in population of Poland

**DOI:** 10.1007/s11033-012-2220-9

**Published:** 2012-10-17

**Authors:** Elżbieta Gałecka, Janusz Szemraj, Małgorzata Bieńkiewicz, Ireneusz Majsterek, Karolina Przybyłowska-Sygut, Piotr Gałecki, Andrzej Lewiński

**Affiliations:** 1 Department of Endocrinology and Metabolic Diseases, Medical University of Łódź, 281/289, Rzgowska St, 93-338 Lodz, Poland; 2 Department of Adult Psychiatry, Medical University of Łódź, Lodz, Poland; 3 Department of Quality Control and Radiological Protection, Medical University of Łódź, Lodz, Poland; 4 Department of Chemistry and Biochemistry, Medical University of Łódź, Lodz, Poland; 5 Department of Medical Biochemistry, Medical University of Łódź, Lodz, Poland; 6 Polish Mother’s Memorial Hospital–Research Institute, Lodz, Poland

**Keywords:** Depressive disorder, Single nucleotide polymorphism, Glucocorticoid receptor

## Abstract

Depressive disorder is a disease characterized by disturbances in the hypothalamo–pituitary–adrenal axis. Abnormalities include the increased level of glucocorticoids (GC) and changes in sensitivity to these hormones. The changes are related to glucocorticoid receptors gene (*NR3C1*) variants. The *NR3C1* gene is suggested to be a candidate gene affecting depressive disorder risk and management. The aim of this study was to investigate polymorphisms within the *NR3C1* gene and their role in the susceptibility to recurrent depressive disorder (rDD). 181 depressive patients and 149 healthy ethnically matched controls were included in the study. Single nucleotide polymorphisms were assessed using polymerase chain reaction/restriction fragment length polymorphism method. Statistical significance between rDD patients and controls was observed for the allele and genotype frequencies at three *loci*: *Bcl*I, N363S, and ER22/23EK. The presence of C allele, CC, and GC genotype of *Bcl*I polymorphism, G allele and GA genotype for N363S and ER22/23EK variants respectively were associated with increased rDD risk. Two haplotypes indicated higher susceptibility for rDD, while haplotype GAG played a protective role with OR_dis_ 0.29 [95 % confidence interval (CI) = 0.13–0.64]. Data generated from this study support the earlier results that genetic variants of the *NR3C1* gene are associated with rDD and suggest further consideration on the possible involvement of these variants in etiology of the disease.

## Introduction

Depressive disorder is one of the most common and disabling psychiatric disorders. For a long time, a link between depression and the endocrine system has not only been suspected but confirmed, as well. The hypothalamic–pituitary–adrenal (HPA) axis is the most important element of the neuroendocrine involved in depression. There are ample evidence for the hyperactivity of HPA axis, altered feedback regulation and increased level of cortisol in unipolar depression [1]. In addition, there are also data informing about the decrease in number of glucocorticoid receptors (GRs) in depressive patients and their increase with antidepressant treatment [2, 3]. In contrast, GRs occurring in high number result in worse response to stress that often brings about depression symptoms [4].

Many peripheral and brain processes are affected by GC and GRs-related signals. These include control of inflammation [5], influence on cognition and synaptic plasticity, as well regulation of stress-response processes [6, 7]. Such results suggest a role of GC and GRs transduction pathways in mood disorders including depression, as the disease is characterized by the increased activity of immune/inflammatory pathways [8], disturbed cognition [9] and synaptic plasticity [10].

HPA-related disturbances, including higher level of cortisol, are not only typical for the patients suffering from depression. They are also characteristic for their first-degree relatives and might be a prodromal risk factor for the developing depression [11]. Moreover, HPA-axis dysfunction and HPA-related genes are associated with immune/inflammatory disorders [12]. There are also data informing that blood cells are resistant to GC [13] and it might possibly participate in the enhancement of inflammatory process.

The findings of the increased cortisol level in depression and the lack of suppression of this hormone with dexametasone prompted to a hypothesis that GRs might have altered sensitivity to GC. Disturbances of many brain related processes involving GRs and increased number of proinflammatory cytokines that influence GRs [14] may explain changes in expression of GRs and sensitivity to GC in depression. In addition, genes involved in the regulation of HPA axis [15] and GR function [16] are related to the risk of depression.

Different sensitivity of GRs and the receptors expression can also depend on the functional condition of the GR and *NR3C1* gene, encoding the molecule. In the healthy population, a group of polymorphisms within the gene for GRs was described. The three mainly known, important single nucleotide polymorphisms (SNPs) include *Bcl*I (rs41423247, C>G), N363S (rs6195, A>G), and ER22/23EK (rs6189 and rs 6190; G>A). The *Bcl*I polymorphism was discovered as a restriction fragment and is determined by a cytosine to guanine substitution at position 647 in intron 2. The sensitivity to GC is in different way associated with that genetic variant. In addition, tissue-specific changes in sensitivity are observed [17–19]. The second variant N363S is located in codon 363 (exon 2). The variant is characterized by asparagine to serine change and is associated with the increased sensitivity to GC [20]. The ER22/ER23 polymorphisms are two linked nucleotide changes in codons 22 and 23. The first variant is silent, and glutamic acid is coded. The second variant results in arginine (R) to lysine(K) change. Presence of the ER22/23EK allele is related to the resistance to GC [21]. Additionally, C-reactive protein concentration [19] and changes in the balance between two isoforms of GR GRα ligand-dependent transcription factor and GRβ—having no transcriptional activity, was found to be related to the polymorphism [22, 23].

Considering the increasing need to replicate and extend genetic studies, we examined the *NR3C1* gene polymorphisms and haplotypes in Polish population. The aim of the present study has been to investigate whether there is an association between polymorphism in gene for GRs and the recurrent depressive disorders (rDD) development.

## Materials and methods

### Subjects

A group of 181 patients, treated for rDD (102 females; 56.4 %), were enrolled into the study. The mean age in that group was 42.6 ± 8.2 years (mean ± SD). The diagnosis was established, according to ICD-10 criteria (F33.0–F33.8) [24]. In all the qualified cases, medical history was obtained, using the standardized composite international diagnostic interview (CIDI) [24]. Additionally, the number of depressive episodes, duration of the disease and the age at onset were assessed in each patient. The control group consisted of 149 healthy subjects (83 females; 55.7 %) with family history negative for psychiatric disorders. The mean age in that group was 38.7 ± 6.7 years (mean ± SD). The control subjects included community volunteers, enrolled to the study following the criteria of the psychiatric CIDI interview [24]. Both patients and controls with other psychiatric diagnoses, concerning axis I and II disorders, were excluded from the study. All the patients and control subjects were native, unrelated inhabitants of the central Poland. An informed consent was obtained from all the participants of the study. The study protocol had earlier been approved by the Local Bioethics Committee No. RNN/626/09/KB.

No significant differences were found between analysed groups with respect to gender (*p* > 0.05). Hardy–Weinberg’s distributions in the individual polymorphisms were as follows: *Bcl*I: polymorphism: for rDD (χ^2^ − 7.08; *p* < 0.0001); for controls (χ^2^ − 2.47; *p* = 0.12); N363S polymorphism: for rDD (χ^2^ − 28.8; *p* < 0.0001); for controls (χ^2^ − 0.26; *p* = 0.61); ER22/23EK polymorphism: for rDD (χ^2^ − 0.41; *p* = 0.52); for controls (χ^2^ − 0.04; *p* = 0.84).

### Genotyping

DNA was extracted from whole blood according to the guanidine thiocyanate (GTC) method. Polymerase chain reaction (PCR)-based restriction fragment length polymorphism (RFLP) for intronic *Bcl*I polymorphism was analyzed using 0.1 μg genomic DNA, 200 μM each dNTP, 5 × Go*Taq* buffer solution, 1 u Go*Taq* polymerase (Promega, Madison WI USA), 0.5 μM specific primers 5′-GAGAAATTCACCCCTACCAAC-3′ and 5′-AGAGCCCTATTCTTCAAACTG-3′. After a 5 min denaturing step amplification was performed, according to the following cycling profile: 94 °C for 30 s, 56 °C for 30 s and 72 °C for 30 s (34 cycles). The final elongation step was 10 min at 72 °C. Amplification product 418 bp was digested with restriction enzyme *Bcl*I (New England BioLabs) at 50 °C for 10 h. The polymorphism was visualized by separating the digested amplification products on 2 % agarose gel. The amplified fragment was 418 bp long from which the restriction reaction yielded fragments of 267 and 151 bp for wild-type homozygous samples, fragments of 418, 267, and 151 bp for wild-type heterozygous samples, and a 418 bp fragment, which was detected in polymorphic (mutant) homozygotes.

Allele-specific PCR for N363S polymorphism was performed with the method designed by Majnik et al. [25]. Allele-specific PCR reaction yielded a control fragment of 357 bp in each tube and a specific fragment of 306 bp in those tubes where the wild type allele (coding asparagine) or the mutant allele (coding serine) corresponding to the applied specific primer was present. Primers sequences were 363 W: 5′-ATCCTTGGCACCTATTCCAAT-3′ corresponding to wild type and 363 M: 5′-ATCCTTGGCACCTATTCCAAC-3′ to mutant Ser allele. The polymorphism was visualized by separating the digested amplification products on 3 % agarose gel.

PCR-based RFLP for exonic ER22/23EK polymorphism was analyzed using 0.1 μg genomic DNA, 200 μM each dNTP, 5 × Go*Taq* buffer solution, 1 u Go*Taq* polymerase (Promega, Madison WI USA), 0.5 μM specific primers 5′-TGCATTCGGAGTTAACTAAAAG-3′ and 5′-ATCCCAGGTCATTTCCCATC-3′. After a 5 min denaturing step amplification was performed according to the following cycling profile: 94 °C for 30 s, 56 °C for 30 s and 72 °C for 30 s (34 cycles). The final elongation step was 10 min at 72 °C. The amplified fragment was 448 bp long and was digested with *Mnl*I restriction enzyme (New England Biolabs) at 37 °C for 4 h. This yielded fragments of 149 and 163 bp (and smaller fragments of 50, 49, and 35 bp) in the presence of the wild-type allele and fragments of 163 and 184 bp (and smaller fragments of 50 and 49 bp) in the presence of the polymorphic allele. The RFLP products were separated and visualized on 3 % agarose gel.

In addition, 10 % of all samples were randomly selected and genotyped in duplicate, and the results were fully concordant.

### Statistical analysis

The results are reported as percentages (%) or means with standard deviations (±SD). In order to examine the association between *NR3C1* gene polymorphisms and rDD, χ^2^ test was used. Post hoc power analysis was performed with the use of non-central χ^2^ distribution. The analysis of association was based on 95 % confidence interval (CI) for the disease odds ratio (OR_dis_), calculated with use of logistic regression model including sex and age as covariates. Departures from the Hardy–Weinberg’s equilibrium were determined by comparison of observed genotype prevalence rates with expected ones. In all the analyses, *p* ≤ 0.05 was accepted as the level of statistical significance.

## Results

Patients clinical characteristics and genotype distribution are shown in Table 1. In both groups, no significant relationships were found between the distributions of analysed genotypes and the demographic variables (age, sex). Relationships were not also observed between analysed genotypes and such clinical variables as the age at disease onset, disease duration and the number of episodes, except for duration of the rDD for N363S polymorphism (Kruskal–Walis’ test; *p* = 0.013) (Table 1).

**Table 1:** Clinical characteristics of patients and genotype distribution

Genotype (*n*)	Age of onset (years)	Duration of the rDD in (years)	Number of episodes
*Bcl*I			
GG (42)	35 ± 5	7 ± 3	4 ± 1
GC (108)	34 ± 6	9 ± 5	4 ± 1
CC (31)	34 ± 6	9 ± 5	4 ± 1
N363S*			
AA (154)	34 ± 6	9 ± 4	4 ± 1
AG (19)	35 ± 6	9 ± 4	4 ± 1
GG (6)	31 ± 2	6 ± 2	3 ± 1
ER22/23EK			
GG (162)	34 ± 6	9 ± 4	4 ± 1
GC (18)	35 ± 5	9 ± 5	4 ± 1
CC (1)	36	7	4

*rDD* recurrent depressive disorders

* Significant differences between genotype distribution and duration of rDD for N363S polymorphism; *n* number of genotypes; Kruskal–Walis test; *p* = 0.013

Data and statistical analyses regarding genotype (heterozygotes or homozygotes) distribution, allele frequency and OR_dis_ values (adjusted for age and sex) determined with respect to the *Bcl*I, N363S and ER22/23EK SNPs of the *NR3C1* gene are presented in Table 2. Significant differences were observed in genotype distribution and allele frequency between rDD-affected patients and controls (Table 2). In all the analysis post hoc power analysis showed high power of testing 1−β = 99 %. When evaluating the *Bcl*I polymorphism, we demonstrated that presence GG homozygote and G allele significantly reduced the risk of rDD while presence of C allele and CC, GC genotypes were a positive factor of the disease risk. The obtained value of OR_dis_ for N363S polymorphism was significant and indicated protective role for A allele while G allele as risk factor for having rDD. Results, regarding the ER22/23EK were demonstrated that a GG heterozygote decreases the risk of rDD while GA increases the risk in question.

**Table 2:** Genotypes and alleles frequencies of the *Bc1*l, N363S and ER22/23EK polymorphisms and risk of rDD

	rDD, *n* (%)	Controls, *n* (%)	OR (95 % CI)	*p*	OR* (95 % CI)	p*
*Bcl*I polymorphism						
Genotypes χ2 = 24.3 *p* = 0.00001						
GG	42 (23.2)	70 (47.0)	0.34 (0.21–0.55)	<0.00001	0.35 (0.22–0.58)	0.00002
GC	108 (59.7)	70 (47.0)	1.67 (1.08–2.59)	0.022	1.66 (1.05–2.62)	0.028
CC	31 (17.1)	9 (6.0)	3.21 (1.47–7.01)	0.003	3.08 (1.38–6.89)	0.006
Allele χ2 = 20.9 *p* < 0.00001						
G	192 (53.0)	210 (70.5)	0.47 (0.34–0.65)	<0.00001	0.465 (0.33–0.65)	<0.00001
C	170 (47.0)	88 (29.5)	2.11 (1.53–2.92)	<0.00001	2.15 (1.53–3.00)	<0.00001
N363S polymorphism						
Genotype χ2 = 7.54 *p* = 0.023						
AA	154 (85.1)	137 (92.0)	0.50 (0.24–1.03)	0.058	0.76 (0.37–1.55)	0.45
AG	19 (10.5)	12 (8.0)	1.34 (0.63–2.86)	0.45	1.32 (0.59–2.91)	0.46
GG	8 (4.4)	–				
Allele χ2 = 7.93 *p* = 0.005						
A	327 (90.3)	286 (96.6)	0.39 (0.20–0.77)	0.0065	0.33 (0.16–0.67)	0.0020
G	35 (9.7)	12 (3.4)	2.56 (1.30–5.00)	0.0065	3.03 (1.49–6.25)	0.0020
ER22/23EK polymorphism						
Genotype χ2 = 6.36 *p* = 0.042						
GG	162 (89.5)	144 (96.6)	0.30 (0.11–0.82)	0.018	0.28 (0.10–0.80)	0.017
GA	18 (9.9)	5 (3.4)	3.18 (1.15–8.82)	0.026	3.39 (1.18–9.75)	0.023
AA	1 (0.6)	–				
Allele χ2 = 6,64 *p* = 0.010						
G	342 (94.5)	293 (98.3)	0.99 (0.70–1.40)	0.95	0.99 (0.71–1.37)	0.95
A	20 (5.5)	5 (1.7)	1.02 (0.71–1.43)	0.95	1.02 (0.73–1.41)	0.95

Odds ratio calculated with the use of logistic regression, *rDD* recurrent depressive disorders, *OR* odds ratio, *CI* confidence interval

* OR—odds ratio age and sex adjusted (with appropriate p* value)

The haplotype analysis results are shown in Table 3. We found five haplotypes with significant differences between cases and controls. Two of them indicate higher susceptibility to rDD with OR_dis_, ranged from 2.16 to 7.48, and one haplotype plays a protective role with OR_dis_ 0.29.

**Table 3:** Haplotypes frequencies of the *Bc1*l, N363S and ER22/23EK polymorphisms

	*Bc1*I	N363S	ER22/23	rDD, *n* (%)	Controls, *n* (%)	OR (95 % CI)	*p*
1	C	A	G	131 (72.4)	79 (53.0)	2.16 (1.34–3.48)	0.0003
2	C	A	A	12 (6.6)	1 (0.7)	7.48 (0.99–56.08)	0.049
3	C	G	G	26 (14.4)	4 (2.7)	7.23 (2.37–22.07)	0.00005
4	C	G	A	12 (6.6)	1 (0.7)	7.48 (0.99–56.08)	0.049
5	G	A	G	148 (81.8)	140 (94.0)	0.29 (0.13–0.64)	0.0022
6	G	A	A	2 (1.1)	5 (3.4)	0.42 (0.08–2.28)	0.31
7	G	G	G	8 (4.4)	11 (7.4)	0.69 (0.26–1.84)	0.45
8	G	G	A	4 (2.2)	4 (2.27)	1.15 (0.27–4.88)	0.84

*rDD* recurrent depressive disorders, *CI* confidence interval, % percentages

*OR* odds ratio adjusted for age and sex by method of logistic regression with 95 % confidence interval given in parentheses; *p* value of Wald statistics with χ^2^ distribution

## Discussion

The main aim of the present study has been to examine, whether genetic variants of *NR3C1* gene are associated with depression, especially with rDD in population of central Poland. The results of our study supported an association between three SNPs of the gene in question, indicating existence of the risky alleles. The haplotype analysis led to the detection of possible variants in which two of them represented vulnerability to rDD.

The relationship between the *NR3G1* gene and depression was observed by other authors [26–28]. In Caucasian population van Rossum et al. [28] found that homozygous carriers of the B*cl*I polymorphism are more susceptible to depression and also found the significant differences in genotype frequency, what is noticeable in our data, as well. Krishnamurthy et al. [29] presented the similar results in a group of depressive premenopausal women. In addition, the presence of B*cl*I polymorphism was associated with lower decrease in Hamilton Rating Scale for depression (HDRS) [30]. An important role for the ER22/23EK carriers and the presence of A allele in patients were observed by van Rossum et al. [28], while we found increased risk of depression for the GA heterozygotes. Here, rather rare presence of AA genotype in Caucasian group should be mentioned. Moreover, post hoc power analysis in our study showed high power of testing. Brouwer et al. [30] results showed a little difference in genotype distribution in depressive Dutch patients (without the differentiation to unipolar depression).

We found increased risk for the G allele for the N363S polymorphism, that is related to increased sensitivity to GC. Higher frequency of G allele was found by van Rossum et al. [28] in German patients suffering from depression. The relation between N362S variant was not observed by van West et al. [26], and by Szczepankiewicz et al. [27] in patients with depression, meeting DSM-IV criteria, while we used ICD-10. Possible source of differences could come from inclusion criteria.

We have also found that haplotype that consists of the major alleles is associated with depression risk. The relation between haplotype of *NR3C1* gene was observed earlier in depression by van Rossum et al. [28] and in patients with coronary heart disease suffering from depression [31].

Data obtained in our study corroborated previous results on involvement of *NR3C1* polymorphisms in depression risk in Caucasian population. Moreover, our results confirmed the need of replication and extension of previous genetic studies. As in earlier studies, we found the relation between rDD and SNPs that probably determined opposite effects of GC and GRs dependent signal transduction pathway. Such results confirm the heterogeneity of rDD etiology and open once again a discussion on the role of GC and GRs in depression management.

According to the results, indicating that N363S variant related to increased sensitivity to GC is a risk factor to depression, one may suggest the role of overactivation of GR and GC related mechanism in etiology of depression. There is an evidence that increased activation of GRs is followed by higher glutamate activity and distortion of neurogenesis—disturbances characteristic to the disease in question. In addition, the increased CRH expression in the brain regions related to depression, as an effect of positive feedback of GC and parallel occurrence of depressive symptoms, should be estimated [32].

ER22/23EK polymorphic allele affecting transcript level is related to the increased resistance to GRs but this allel increase the risk of depression, as well. In this case it appears as if the GC and GRs were important molecules for depression. The decrease in GRs expression and resistance to GC may result in increase of inflammatory activity, observed in depression [33]. ER22/23EK polymorphism seems to be modestly associated with a decreased risk for dementia, the latter is claimed to be connected with depression [34]. One may hypothesize and may suggest the important role of genotype dependent GR mechanism in depression, as in the inflammatory disease. Inflammatory nature of depressive disorder suggests that GC and GRs related signal plays a role in the disease susceptibility. According to Kuningas et al. [35] ER22/23EK variant is related to higher C-reactive protein level in patients with cardiovascular disease that often co-exists with depression. Van Vinsen et al. [36] demonstrates sensitivity of monocytes, that activity and number are increased in depression [37], in carriers of the mutant allel G of N363s variant. Moreover, polymorphic variant of *Bcl*I, associated with the reduced sensitivity of leucocytes to GC [17] is according to our data a risk factor for rDD. Allel G and GG homozygote was also found to be involved in higher inflammatory activity [12]. Above results support that inflammatory process in depression might be related to GS and GRs signal. Regarding the *Bcl*I variant, it should be considered that this polymorphism is intronic and has no functionality or is linked with functionally important variant. The *Bcl*I variant was not found to be associated with the depression risk in patients meeting DSM-IV criteria [27]. The polymorphism tissue specificity is also important. Nevertheless, in several inflammatory diseases, decreased sensitivity of blood cells to GC was observed [38, 39]. The role of other inflammatory—related genes cannot be excluded. Moreover, there are other genotype dependent and independent mechanisms, influencing the GR sensitivity. For example, changes in sensitivity might be determined by other gene variants [40]. There is also evidence for influence of interferon beta, cytokines and macrophage migration inhibitory factor on GR sensitivity and function [36, 41, 42].

## Conclusion

The genetic variants, which are associated with GC sensitivity, may belong to factors leading to the depression development. The present study addressed and confirmed the importance of *NR3C1* gene polymorphism in rDD, especially in Caucasian population. The findings suggest that not only the increase but also the decrease in GRs related mechanism can participate in induction of depression. The opposite results emphasize the wide and multifactorial role of GC and GRs and suggest that the GRs-related transduction pathway and its effect should be taken into account, considering, considering various cells and different conditions.
